# Utilization Perspectives of Lignin Biochar from Industrial Biomass Residue

**DOI:** 10.3390/molecules28124842

**Published:** 2023-06-18

**Authors:** Iliyana Naydenova, Temenuzhka Radoykova, Tsvetelina Petrova, Ognyan Sandov, Ivo Valchev

**Affiliations:** 1 Department of Energy and Mechanical Engineering, Technical College-Sofia, Technical University of Sofia, 1000 Sofia, Bulgaria; 2 Department of Pulp, Paper and Printing Arts, Faculty of Chemical Technologies, University of Chemical Technology and Metallurgy, 1000 Sofia, Bulgaria

**Keywords:** technically hydrolyzed lignin, carbonization, biochar characterization

## Abstract

The present study aimed at utilizing technically hydrolyzed lignin (THL), industrial biomass residue, derived in high-temperature diluted sulfuric acid hydrolysis of softwood and hardwood chips to sugars. The THL was carbonized in a horizontal tube furnace at atmospheric pressure, in inert atmosphere and at three different temperatures (500, 600, and 700 °C). Biochar chemical composition was investigated along with its HHV, thermal stability (thermogravimetric analysis), and textural properties. Surface area and pore volume were measured with nitrogen physisorption analysis often named upon Brunauer–Emmett–Teller (BET). Increasing the carbonization temperature reduced volatile organic compounds (40 ÷ 96 wt. %), increased fixed carbon (2.11 to 3.68 times the wt. % of fixed carbon in THL), ash, and C-content. Moreover, H and O were reduced, while N- and S-content were below the detection limit. This suggested biochar application as solid biofuel. The biochar Fourier-transform infrared (FTIR) spectra revealed that the functional groups were gradually lost, thus forming materials having merely polycyclic aromatic structures and high condensation rate. The biochar obtained at 600 and 700 °C proved having properties typical for microporous adsorbents, suitable for selective adsorption purposes. Based on the latest observations, another biochar application was proposed—as a catalyst.

## 1. Introduction

Biomass residues are generated annually in huge amounts as a result of different human activities. The plants’ structure contains three main components that are in different proportions—cellulose, hemicellulose, and lignin. Generally, the cellulose content is predominant, followed by lignin [1]. Actually, lignin is the main by-product obtained in a plentiful amount from numerous industrial processes [2], such as the food and paper industries, lignocellulose-based biorefinery, etc. [3,4,5]. For example, only the pulping industry generates around 40 million tons of lignin annually [2]. Due to its calorific value, lignin is often used as solid biofuel in industrial boilers. However, lignin has the potential as feedstock that substitutes the petroleum-based products utilized to manufacture industrial coatings, gels, emulsifiers, etc. [5,6]. In fact, lignocellulose biomass is considered a major source of value-added products and a bioenergy carrier worldwide [7].

In recent decades, the utilization of lignocellulosic matter as a source of renewable fuel, chemicals, or porous biochar derivatives is gaining considerable attention due to its neutral carbon cycle [8]. Comprehensive utilization of lignocellulosic biomass is possible after solving the issue related to its decomposition. The bio-refinery might be more effective if, along with ethanol production from cellulose and hemicellulose, the factory succeeds to obtain value-added products also from the rested hemicellulose and lignin [5]. On the other hand, lignin has been widely studied and typically processed for producing bio-based fuels and chemicals [9]. The significant interest in ethanol production from vegetal raw materials places the question of adequate utilization of the resulting biomass residue. The large internal surface of the lignocellulosic material and the availability of different functional groups could suggest the possible usage of such materials as adsorbents of metal ions, e.g., for water purification purposes. Renewable agricultural residues are produced in bulk as waste, and their storage and management create an environmental problem. The application of agricultural wastes as biosorbents is possible directly or after activation [10,11,12]. Previous investigations [13] proved that some of the biosorbents’ advantages are biodegradability and good adsorption properties due to their morphology and surface functional groups distribution.

Biochar is often generated from lignocellulosic biomass residue by applying a thermal conversion technique, such as gasification, pyrolysis, torrefaction, carbonization, or hydrothermal liquefaction [14,15]. 

Pyrolysis is a process of thermal degradation at a limited amount of oxygen and the initial lignocellulosic material can be transformed into solid, liquid, and gaseous products [16]. The pyrolysis-based technologies show great promise for converting lignin and other wood components into biochemicals, biomaterials, and biofuels [17]. The process can be divided into fast and slow pyrolysis, which give different yields of the desirable products [18]. Arni [18] examines the yield of gaseous products during both fast and slow pyrolysis of lignocellulosic feedstock (sugarcane bagasse). The findings are that the low temperature is a better condition for producing methane other than hydrogen for both processes, while high temperature aids in obtaining hydrogen. 

Carbonization is a slow pyrolysis technology in which the processed biomass is heated and turned into biochar after thermal decomposition under inert conditions [19]. 

The valorization of lignin can be performed via its chemical modification to obtain bioactive derivatives, i.e., sulfated lignin, which has anticoagulant and antiplatelet activity and can be used in the treatment of thrombotic disorders [20]. The authors optimize experimentally and numerically the process of sulfation of ethanol lignin birch wood with a mixture of sulfamic acid and urea in a 1,4-dioxane medium. The aim is to characterize the structure and thermochemical properties of the sulfated ethanol lignin. The findings are that obtained sulfated birch ethanol lignin has properties for use in the production of new sorbents, biocomposites, and nanomaterials, as well as in the development of new anticoagulant and antiviral medicines. Kazachenko et al. [21] examined the effect of a type of solid acid catalyst on the sulfation of wheat straw soda lignin with sulfamic acid in a 1,4-dioxane medium, to elucidate the possibility of recycling and to examine the composition and structure of the obtained products. The conclusions are that the solid catalysts used in the sulfation process cause hydrolysis reactions and reduce the molecular weight and polydispersity index.

Hydrogenation is an efficient and reliable technology for lignin valorization, aiming to diminish the difficulties, related to the extraction of its functional phenolic compound [22]. Abdullah et al. [23] present lignin hydrogenation as a depolymerization method, which uses hydrogen as a reductant under mild conditions. The authors aim at obtaining aromatic products with low oxygen content and increased products’ stability. The most critical decision for such processes is the selection of suitable catalysts [24,25]. The phenolic compounds can be increased using the catalytic liquefaction reactions, and such an example is well described in [26], testing various bimetal selective catalysts and alcoholic solvents. 

Currently, a great research effort is imposed to establish barely studied and effective materials for the disposal of harmful/pathogenic elements or organisms in the air and water environment. In this respect, activated carbon has emerged as promising material. Activated carbon has been used for such purposes as a stand-alone material or as a carrier of active ingredients. However, the requirements and regulations for its production are continuously increasing, especially in terms of its porous texture. It is necessary to create the structure with pores of a specific size, which would increase the material’s selectivity in relation to certain components that need to be removed. Various types of feedstocks are used to produce activated carbon. When waste matter is utilized, it reduces the feedstock’s cost and solves related environmental problems. In view of this, technically hydrolyzed lignin (THL) is of particular interest because it is typically generated in large quantities as a hardly utilized by-product of certain industrial processes. According to Shiraki et al. [27], the concentrated sulfuric acid can completely swell and hydrolyze cellulose. The authors consider the concentrated sulfuric acid hydrolysis as the most effective process capable of recovering the maximum yield of monomeric sugars from woody biomass. Further, they discuss the difficulties of utilizing the solid by-product (lignin) because of the self-condensation between the lignin molecules under acidic conditions and propose a method for lignin valorization using a unique additive, t-butyl alcohol. The results show unchanged sugar yields along with a lignin yield higher than 40%. Thermoplastic lignin with good solubility is successfully recovered in acetone, and the method is foreseen as a new candidate for implementation in sugar platform biorefineries.

The present work aimed at investigating a utilization path for industrial biomass residue, namely technically hydrolyzed lignin (THL). In the area of Razlog, Republic of Bulgaria, a 140-acre landfill of hydrolysis lignin residue is located. The THL had been deposed outdoor for many years, and its total amount is evaluated to be about 350,000–400,000 tons. This THL was formed as by-product of a high-temperature diluted sulfuric acid hydrolysis of softwood and hardwood chips to sugars, which were further subjected to yeast fodder production. The accumulated huge amount of THL releases different gaseous air pollutants including greenhouse emissions. During the summer period, when the outdoor temperature significantly increases, this matter is also self-igniting. Therefore, the present case study aims to propose a THL utilization method. This type of biomass residue is of potential harm to the surrounding environment and population. For that purpose, the THL was carbonized in a horizontal tube furnace (HTF) at a temperature range between 500 and 700 °C. The experimental set up is described elsewhere [19,28]. The obtained biochar was chemically characterized through a set of chemical and physical analyses [13,29,30,31]. The possible biochar applications were discussed in line with the present European effort for circular economy and climate change preservation (e.g., Regulation (EU) 2018/1999 and Directive (EU) 2018/2001). The suggested methodology was based on a well-established technique for THL thermal conversion and methods for the product’s characterization. It provides the basis for detailed investigations nationwide on both optimized biomass conversion and products utilization, thus reducing the negative footprint of the local biorefineries.

## 2. Results and Discussion

### 2.1. Effect of Carbonization Temperature on Biochar Yield and Its Chemical Composition

Several analytical methods were used (proximate, ultimate, ash, calorimetric, and lignocellulosic analyses) to characterize both THL and biochar. The obtained results were summarized in Table 1. Increasing the carbonization temperature led to significantly reduced content of volatile organic compounds (from 40 to 96 wt. %) and increased fixed carbon (from 2.11 to 3.68 times the FC wt. % in THL) and ash content. Farrokh et al. [32] report similar effects. The authors examine lignin biochar produced at three different temperatures (300, 500, and 650 °C). In addition, H- and O-content was considerably reduced along with the N- and S-content, which for some samples was measured below the detection limit. The results from the ultimate analysis are in accordance with [32,33]. However, the higher heating value (HHV) and the biochar yield slightly decreased with increasing the carbonization temperature, due to the structural transformations in the carbonization process, relevant to the chosen experimental conditions. The effect was observed also in [34], concerning biochar samples, obtained at temperatures above 500 °C. The results were in line with the investigations of [35]. The authors proved that increasing the carbonization temperature and/or residence time often leads to lower biochar mass yield and HHV.

The lignocellulosic analysis confirmed that during the diluted sulfuric acid hydrolysis of the initial biomass, the hemicellulose was hydrolyzed and the THL became rich in lignin and resistant to hydrolysis cellulose fraction.

The ICP-OES spectroscopy allowed determining the ash composition of THL and its carbonized products. Table 2 summarizes the mean values from three independent repetitions of each experiment. Except for Pb, Si, and Na, the rest of the elements were concentrated in the biochar, showing significant temperature dependence. According to [34], increasing the carbonization temperature might lead to the volatilization of some metals. Expectedly, increasing the carbonization temperature led to a higher concentration of most of the measured elements in the biochar, generated at 700 °C.

### 2.2. Thermal Analysis

Thermal stability analyses, such as Thermogravimetric (TG), Differential Thermal Analysis (DTA), and Differential Scanning Calorimetry (DSC) are typically used to estimate the processes of thermal degradation of biomass and its derivatives. 

**Figure 1:**
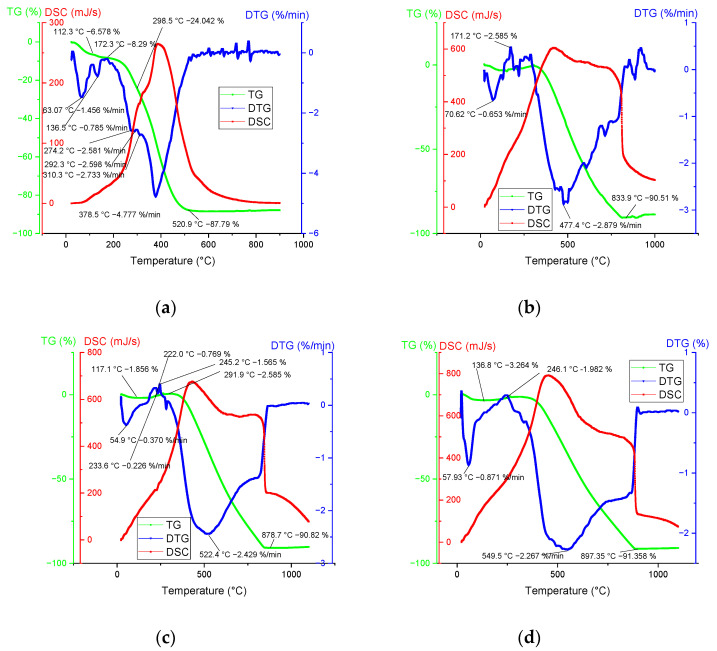
Thermal analysis of THL (**a**) and biochar, obtained at 500 °C (**b**), 600 °C (**c**), and 700 °C (**d**) in dynamic heating mode (20 ÷ 1000 °C), and constant heating (10 K/min) and air flow (100 mL/min) rates.

Herein, simultaneous TG-DTA/DSC study was carried out, and the thermal conversion of THL and biochar (derived at 500, 600, and 700 °C) was investigated along with the effects of weight loss and thermal stability. The graphic interpretation of the TG-DTG-DSC temperature dependence is illustrated in Figure 1. The following three global stages were identified:

- Stage 1—Water vaporization was determined in the temperature range between room temperature (RT) and 246 °C. Typical for this stage, an endothermic peak was observed, which normally corresponds to the elimination of humidity, followed by broad exothermal peaks.
- Stage 2—Devolatilization and dehydrogenation (of some hydroxides in the mineral composition) took place in the following temperature range: 175 ÷ 900 °C.
- Stage 3—Fixed carbon combustion was observed at temperatures between 520 and 950 °C. The TGA curves of biochar showed that this stage overlapped with stage 2.

The lignin decomposes slower and over a broader temperature range [37] in comparison to cellulose and hemicellulose [38]. The effect is attributed to the specific thermal stability of some oxygen-containing functional groups with scission occurring at lower temperatures [39].

As expected, the present thermal analyses showed the occurrence of mostly exothermic reactions. The DSC peaks coincided well with the appearance of the maximum mass loss rates (Table 3). The peaks at higher temperatures were associated with the thermal decomposition of both lignin and difficult to hydrolyze polysaccharides [38].

The complex decomposition of THL (see, e.g., its DTG curve in Figure 1 and Table 3) resulted in at least five overlapped steps with maximum mass loss rate at 378.5 °C and a long tail beyond 500 °C. Instead of one simple peak at 300 °C, the THL showed a complex destruction process between 270 and 310 °C, which was related to cellulose degradation [40]. 

The maximum mass loss rate of THL (4.78 %/min) was observed at 378.5 °C. This behavior was related to the low cellulose content in the examined material (Table 1). The THL thermal decomposition finishes at about 530 °C. The biochar degradation showed that increasing the carbonization temperature broadened the interval to the total decomposition from 835 °C (biochar, obtained at 500 °C) to 895 °C (biochar—at 700 °C) as well as increased the peak temperature in the same order.

### 2.3. Fourier-Transform Infrared (FTIR) Spectroscopy

**Figure 2:**
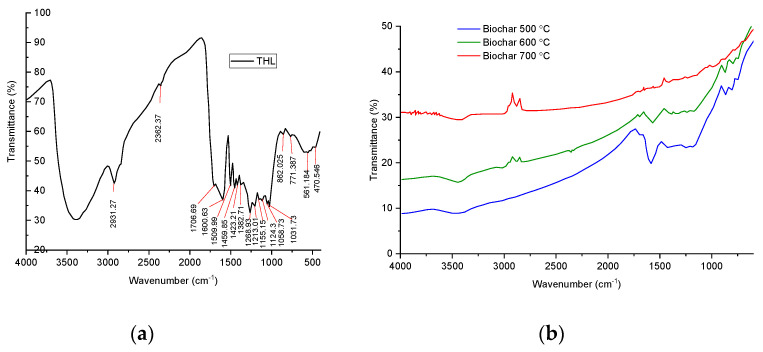
FTIR spectrum of THL (**a**) and the biochar, derived at 500 °C, 600 °C, and 700 °C (**b**).

The effect of temperature on the functional groups of THL and its carbonized products was studied also with FTIR spectroscopy (Figure 2). The FTIR spectrum of THL was influenced by the higher content of lignin and polysaccharides. The wide band at 3400 cm^−1^, belonging to the zone 3500–3100 cm^−1^, was due to the valence vibrations of alcoholic (phenolic) and hydroxyl groups included in hydrogen bonds [41]. The intensive bands at 2931 and 2800 cm^−1^ referred to different types of valence vibrations of CH bonds in the methyl and methylene groups. The band at 1710 cm^−1^, falling in the range 1600–1760 cm^−1^, was characteristic of the vibrational oscillation of the group C=O in alkyl-aromatic ketones. In particular, a ketocarbonyl group is typically supported by β-carbon atom of a propane chain [42]. The bands at 1600 and 1509 cm^−1^ were associated with vibrations of aromatic nuclei [43]. The bands at 1459 cm^−1^ and 1382 cm^−1^ denote deformation vibrations of CH in the methyl and methylene groups, while the band at 1155 cm^−1^ referred to C-O-C asymmetric vibrational oscillation in ether groups. Further investigation of the THL–FTIR spectrum attributed the 862 cm^−1^ band to deformation vibrations of the CH bonds in a three-substituted aromatic nucleus, and the one at 771 cm^−1^ was due to deformation vibrations of the CH bonds in a mono-substituted aromatic nucleus [43].

The FTIR spectra of biochar proved that their functional groups were gradually lost with increasing the carbonization temperature, where the role of the polycyclic aromatic structures was significant [34]. This conclusion is in line with the observations, reported by [44]. The authors discussed that such results are helping to explore the applicability of different types of biochar for the immobilization of specific environmental pollutants, carbon sequestration, etc.

The band at 3443 cm^−1^ referred to O–H stretching of H-bonded hydroxyl groups, while the one at 2870 cm^−1^ was ascribed to symmetric C–H stretching of aliphatic hydrocarbon (e.g., from the propane chain of the monomer units in lignin). The band at 1695 cm^−1^ referred to C=O stretching vibrations of alkyl-aromatic ketones, whereas the bands at 1600 cm^−1^ and 1430 cm^−1^ were connected with C=C stretching vibrations of aromatic components. The results correspond well with Li et al. [45]. A similar observation is reported by Wang et al. [46] reporting the split of the phenolic groups at temperatures above 500 °C. The bands at 870 cm^−1^, 811 cm^−1^, and 757 cm^−1^ were due to C–H bending vibrations from three-substituted, di-substituted, and mono-substituted aromatic nuclei, respectively [43]. The band at 1191 cm^−1^ was attributed to C–O–C symmetric stretching vibrations in ester groups, while the bands, detected between 870 cm^−1^ and 675 cm^−1^ were associated with C–H bending vibrations [45].

### 2.4. Surface Area and Pore Volume

**Figure 3:**
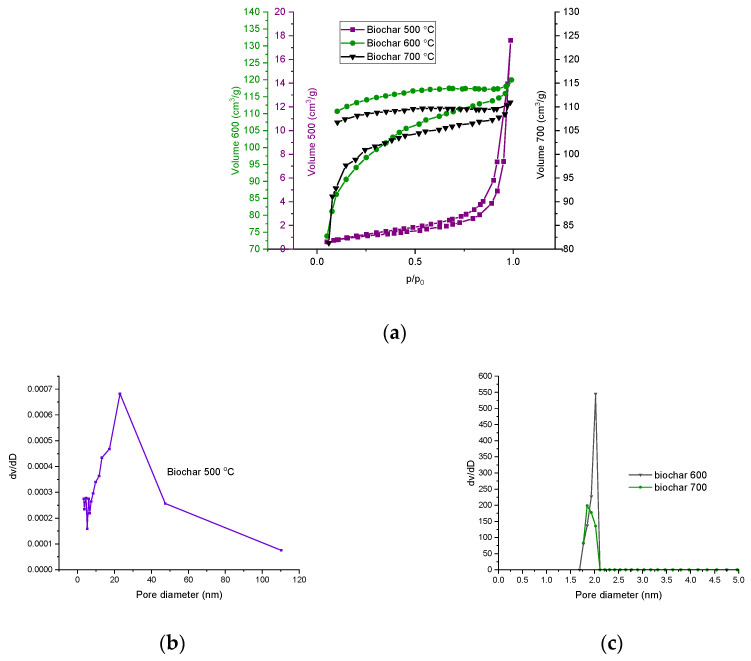
(**a**) Nitrogen adsorption–desorption isotherms and pore size distribution of (**b**) biochar—500 °C and (**c**) biochar—600 and 700 °C.

The evaluation of the specific surface area of the THL and biochar was carried out by adsorption of nitrogen at −196 °C. Nitrogen adsorption–desorption isotherms were used to calculate the specific surface area using the BET equation [13]. The results are summarized in Table 4 and Figure 3. 

The isotherm of THL is of type II, according to IUPAC classification, evidencing the material is nonporous or microporous (Figure 3). The hysteresis loop is of type H3, which could be attributed to aggregates of plate-like particles giving rise to slit-shaped pores.

The isotherms of the samples of biochar are of type I, indicating that the micropores were dominating the textural properties of the biochar derived at 600 and 700 °C. The hysteresis loop of H4 type herein started at relatively higher pressure, due to which two types of pores were considered: mesoporous and microporous. The H4 loop is often attributed to narrow slit-like pores. The hysteresis loops do not close for the biochar samples, derived at 600 and 700 °C. This could be due to hindered evaporation of the trapped nitrogen because of the heterogeneous coal surface or ink-bottled pores [47,48]. These results were confirmed by the specific surface area estimations. The BET equation, used for determining the surface area, was applied in the interval of relative pressure (P/Po) between 0.05 and 0.35, considering partial surface occupation. The BET surface area obtained in the present work is in line with the data reported in earlier investigations of Wang et al. [46] on the characterized biochar, produced from (bamboo and elm) woody residue, pyrolyzed at 500 or 700 °C. They confirm that increasing the carbonization temperature results in an increased BET surface area. Similar temperature dependence was reported also by Shaaban et al. [49] in their characterization of biochar, derived during slow pyrolysis of rubber wood sawdust (300–700 °C). The pore size distribution was also plotted for all examined biochar samples and generally confirmed the results for the porous texture, which were deduced from the adsorption isotherms. The adsorption isotherms of the samples obtained at 500 °C denote that the formation of micropores began at this temperature but the mesopores dominated over the micropores. The biochar, obtained at 600 and 700 °C showed a narrower interval of pore-diameter variations (1 ÷ 2 nm). Thus, an opportunity is foreseen for selective adsorption of molecules, having particular size and/or chemical structure, typical for some microporous adsorbents.

## 3. Materials and Methods

### 3.1. Feedstock Origin

The investigated THL was a typical example of industrial biomass residue, derived during high-temperature diluted sulfuric acid hydrolysis of softwood and hardwood chips to sugars. In order to develop an efficient biomass processing technology, it is crucial to understand its characteristics and decomposition behavior. Herein, the THL was carbonized at well-controlled conditions.

### 3.2. Experimental Equipment

**Figure 4:**
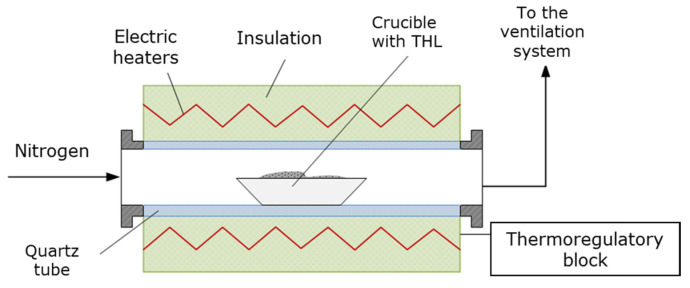
Schematic interpretation of the experimental setup—HTF.

The THL was carbonized in HTF (Figure 4) at atmospheric pressure and at three different temperatures, 500, 600, and 700 °C. The residence time of a single sample within the reaction zone was one hour [19]. The carbonization process was carried out in inert atmosphere (nitrogen), with nitrogen flow rate of 1 L/min, and heating rate of 24 °C/min. The HTF was thoroughly described elsewhere [28].

At the end of the process the crucibles, containing biochar, were covered and tempered in a desiccator for at least an hour. Then, the samples were weighed with an analytical balance. Thus, the *biochar mass yield* was obtained according to the following equation:

(1) $$\mathrm{Biochar}\mathrm{mass}\mathrm{yield}=\frac{mass\;of\;biochar\;\left(g\right)}{mass\;of\;THL\;\left(g\right)}\cdot 100,\;wt.\%,$$

### 3.3. Feedstock and Biochar Characterization

The feedstock (THL) and the obtained biochar were chemically characterized through proximate, ultimate, ash, lignocellulosic, and calorimetric analyses. The lignocellulosic composition of the THL was determined according to the following methods: cellulose [29] and lignin [30].

The ultimate analysis (C, N, S, and H) of all types of samples was performed with an Elemental Analyzer Eurovector EA 3000.

Inductively Coupled Plasma Optical Emission Spectroscopy (ICP-OES) was applied for the ash analysis of both THL and its carbonized products. The analysis was performed by pre-acid decomposition, and the elemental content was evaluated by Prodigy High Dispersion ICP-OES, Telledyne Leeman Labs using US and BDS EN ISO 11885:2009 Standard [31].

In the present work, simultaneous thermal analyses were carried out with STA PT 1600 TG-DTA/DSC analyzer (LINSEIS Messgeräte GmbH, Germany) in dynamic heating mode from room T (RT = 20 °C) to 1000 °C, with constant heating (10 °C/min) and air flow rates (100 mL/min) and in static oxidizing conditions (still air).

The biochar was examined through Fourier-transform infrared (FTIR) spectroscopy, as well as thermal and nitrogen physisorption analysis. The FTIR spectroscopy was carried out using Varian 660 IR spectrometer. The infrared spectra were collected in the mid-infrared region (4000–400 cm^−1^). The samples were prepared by the standard KBr pellets method. The specific surface area of the biochar was determined by low-temperature (77.4 K) nitrogen adsorption in a Quantachrome Instruments NOVA 1200e (USA) apparatus. Before the analyses, the samples were outgassed (argon) at 120 °C for 16 h in a vacuum. The nitrogen adsorption–desorption isotherms were used to evaluate the following parameters: the specific surface area (SBET) was determined through the Brunauer, Emmett, and Teller (BET) equation [13]; the total pore volume (Vt) was estimated in accordance with the Gurvich rule at a relative pressure close to 0.99; the volume of the micropores (V_MI_) and the specific surface area connected to micropores (S_MI_), as well as the external specific surface area (S_EXT_), were evaluated according to V–t-method; additionally, the pore size distributions (PSD) were calculated by equilibrium nonlocal density functional theory (NLDFT) method using slit shape kernel for carbons.

## 4. Conclusions

In the present study, technically hydrolyzed lignin was utilized, and the effect of carbonization temperature on the physicochemical properties of biochar was experimentally measured. Increasing the carbonization temperature (500 ÷ 700 °C) led to the following general conclusions, in view of which the possible use of biochar was proposed, as follows:

- The biochar mass yield slightly decreased with increasing the carbonization temperature. The chemical characterization showed biochar with gradually reduced content of volatile matter, between 40 and 96 wt. % in contrast to THL. The fixed carbon content was increased from 2.11 to 3.68 times the wt. % of fixed carbon in the THL, along with slightly increased ash content. Besides Pb, Si, and Na, most of the elements showed increased concentration in the biochar ash, with increasing the carbonization temperatures. As expected, the ultimate analysis showed significant increase in the C-content, but considerably reduced H- and O-composition, whereas the reduction of the N- and S-content in the high-temperature biochar showed values below the detection limit. This suggested possible biochar application as solid biofuel as well as for soil amendment (e.g., as compensatory fertilizer for trace elements) as discussed in [50].
- The textural analysis (FTIR spectroscopy) showed that the functional groups were gradually lost thus, forming materials characterized merely by polycyclic aromatic structures and high condensation rate.
- The results from the nitrogen physisorption analysis along with those from the FTIR spectroscopy suggested that the proposed utilization technology of THL (specifically the carbonization at 600 and 700 °C) produced biochar, having the properties typical for the microporous adsorbents, which allows for selective adsorption of specific molecules. Based on the latest observations, another possible application was assumed—as a catalyst.

The perspective proposed herein for THL covers a narrow line of opportunities generally based on the applied conversion technique and characterization methods. According to Ramos et al. [51], at present, about 60% of the lignin (obtained often by kraft pulping) is utilized for heat and chemicals in large-scale industries. The authors summarized earlier investigations on lignin utilization where depending on the chosen conversion process [52] lignin can be converted to a great variety of valuable chemicals and materials, such as: (a) hydrocarbons, phenols and catechols, benzylic aldehydes, quinones, alkyl benzenes, bio-oil, carbon fibers [53]; (b) activated carbon and polymeric materials such as phenol-formaldehyde resins, which is precursor for carbon fibers production [54]; (c) highly functionalized molecules, such as phenolic aldehydes, phenolic ketones, phenolic acids, and many others [20,51]. 

In view of the extensive research carried out during the last decades and the current legislation framework, it is more than ever time to stimulate the Member States to consider deposited lignocellulosic residue as a valuable resource of goods, which least existence is the natural degradation and the related environmental concern.

## Figures and Tables

**Table 1:** Chemical characteristics of the samples.

Parameter Studied	THL ^2^	Biochar 500 °C	Biochar 600 °C	Biochar 700 °C
Proximate analysis, wt. %				
Volatiles, db	65.27	38.36	6.42	2.16
Fixed carbon, db ^1^	23.34	49.32	81.41	85.99
Moisture	7.78	4.98	3.74	3.48
Ash, db	3.61	7.35	8.00	8.37
Ultimate analysis, wt. %, db				
C	55.54	76.70	83.72	85.39
H	7.10	3.5	2.65	1.64
N	0.26	-	-	-
S	0.74	0.05	0.05	-
O ^1^	24.97	7.42	1.84	1.12
HHV, db, MJ/kg	23.27	31.36	29.17	29.20
Lignocellulosic analysis, wt. %, db				
Cellulose	25.5	-	-	-
Lignin	72.6	-	-	-
Mineral substances	2.8	-	-	-
Cellulose	25.5	-	-	-
Biochar mass yield, wt. %	-	42.95	40.10	37.99

^1^ By difference; ^2^ Reported in [36].

**Table 2:** Ash composition of THL and biochar.

Chemical Elements, g/kg	THL	Biochar 500 °C	Biochar 700 °C
Al	1.329	4.045	4.860
Ba	0.069	0.135	0.132
Ca	1.790	3.937	4.267
Cu	0.028	0.047	0.049
Fe	0.362	0.729	0.807
Pb	0.002	<0.01	<0.01
Mg	0.142	0.324	0.354
Mn	0.010	0.020	0.025
K	0.378	1.330	1.458
Na	0.093	0.128	0.058
Sr	0.016	0.047	0.049
Zn	0.006	0.014	<0.01
Si	0.138	0.041	0.074
C	<0.01	<0.01	<0.01
Ti	<0.01	<0.01	<0.01
S	0.587	1.776	1.680

**Table 3:** Thermal characteristics of the studied materials.

Stage	Mass Loss	Temperature at Max Loss Rate	Max Mass Loss Rate	Total Mass Loss	Heat Effect
No.	wt. %	°C	%/min	wt. %	MJ/kg
THL					
1	6.58	63.7	1.456	87.79	26.48
2	1.71	136.5	0.785		
3	79.5	378.5	4.777		
Biochar 500 °C					
1	2.59	70.62	0.653	90.51	73.28
2	87.9	477.4	2.879		
Biochar 600 °C					
1	1.6	54.9	0.370	90.82	74.01
2	89.22	522.4	2.429		
Biochar 700 °C					
1	3.26	57.93	0.871	91.36	87.59
2	88.1	549.5	2.267		

**Table 4:** Basic adsorption-textural parameters of THL and biochar.

Sample	S_BET_, m^2^/g	V_t_, cm^3^/g	V_MI_, cm^3^/g	D_AV_, nm	S_MI_, m^2^/g	S_EXT_, m^2^/g
THL	4	0.03	-	28	-	-
Biochar 600 °C	378	0.19	0.10	2.0	267	111
Biochar 700 °C	430	0.17	0.13	1.6	383	47

## Sample Availability

Not available.
